# Supplementary material for: Prolonged exposure to insulin might cause epigenetic alteration leading to insulin resistance
Source: FEBS Open Bio. 2024 Oct 29;15(1):81–93. doi: 10.1002/2211-5463.13891 (PMC11705401; doi:10.1002/2211-5463.13891)
Supplement: Supplementary file 1 — Fig. S1. Immunoblotting to assess AKT phosphorylation status in insulin‐resistant cells. Fig. S2. Gene ontology analysis of differentially expressed genes in insulin‐sensitive and insulin‐resistant BRL‐3A cells. Fig. S3. Protein–protein interaction network associated with IR. Fig. S4. Animal IR model. [file FEB4-15-81-s001.pdf]

A

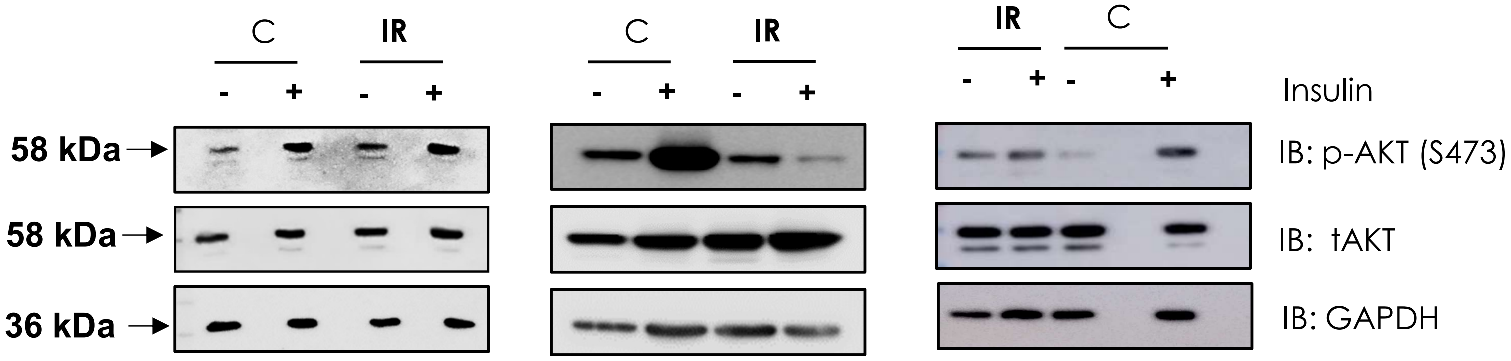

B

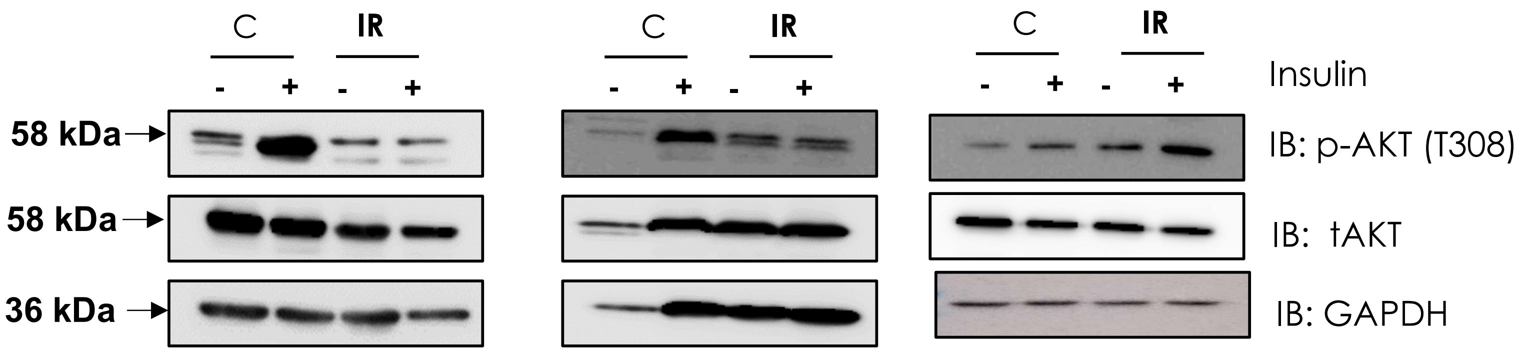

Supplementary Figure S1. Immunoblotting to assess AKT phosphorylation status in insulin resistant cells . A-B: The phosphorylation of Akt was assessed in IR and C cells by probing for phosphorylated Akt, total Akt and GAPDH**.**

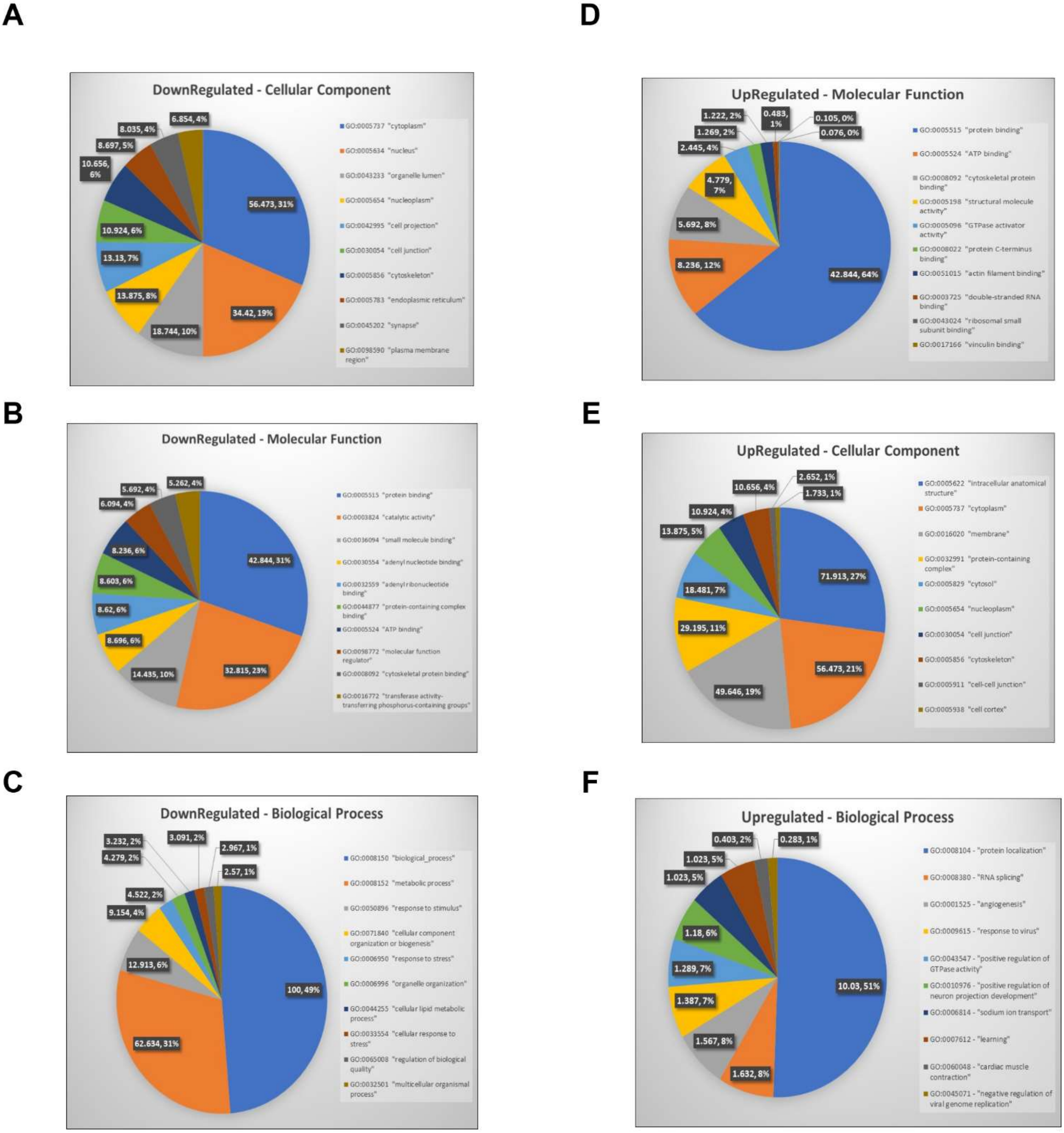

Supplementary Figure S2. Gene ontology analysis of differentially expressed genes in insulin sensitive and resistant BRL-3A cells. A-F: represents significant GO analysis, where highlighting the cellular component, molecular function and biological process (FDR adjusted P-values <0.05), in BRL-IR vs BRL -control cells.

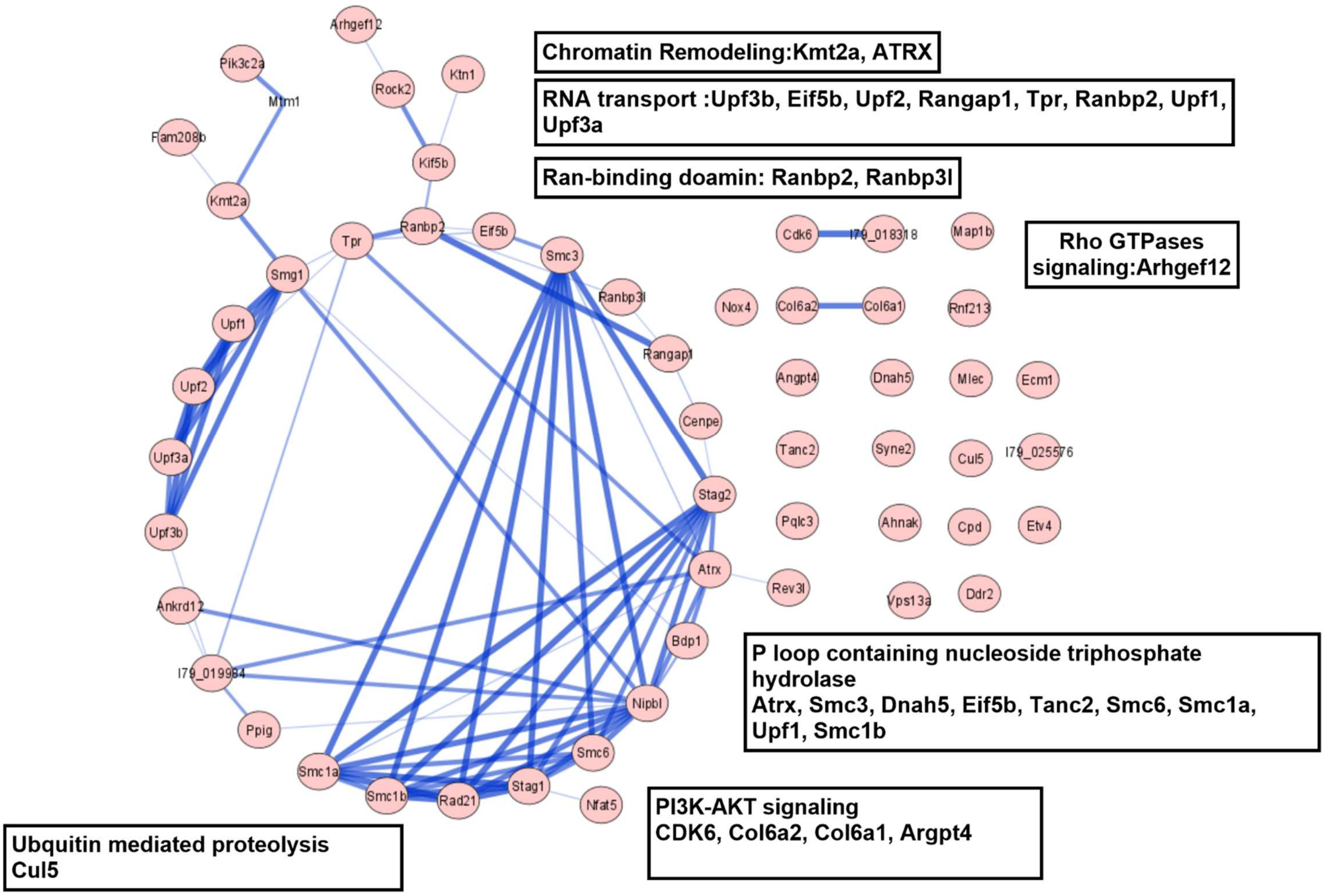

Supplementary Figure S3. Protein-protein interaction network associated with IR. Network of predicted protein-protein interactions from STRING analysis (Szklarczyk et al., 2017) using insulin resistance regulated genes in CHO-GLUT4 IR cells.

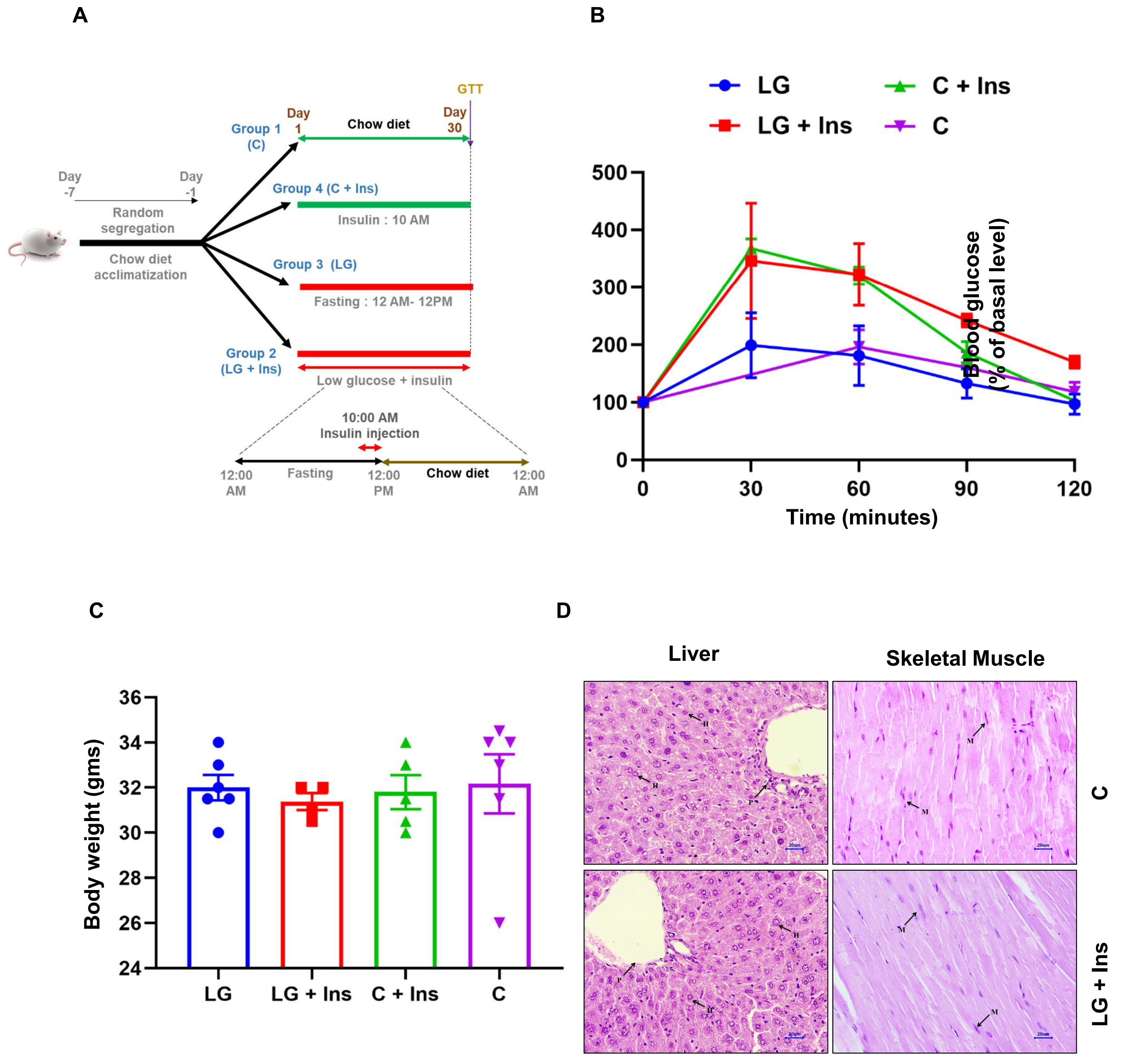

Supplementary Figure S4. Animal IR model. A: Schematic workflow of development of mouse model of IR. **B**: Glucose tolerance test after four weeks of post treatment: Plasma glucose concentrations during the intraperitoneal glucose test (IPGTT;3g/kg body weight) following 8 h of fasting in Swiss Albino male mice were estimated at various time points and graph was plotted for the same. **C:** Body weight at day 30 of all the groups. **D:** Haematoxylin and eosin staining from mice tissue. All images are presented at a magnification of x400. The upper and lower panel represent histology of tissue taken from C and IR group, respectively; where H stands for hepatocyte and P stands for portal triad; where M stands for myocyte. The data represents mean±SEM; n=3-6 and students t test was performed to assess the statistical significance of the data.
